# Exploration of Strawberry Fruit Quality During Harvest Season Under a Semi-Forcing Culture with Plants Nursed Without Chilling

**DOI:** 10.3390/plants13213052

**Published:** 2024-10-31

**Authors:** Paula Pedrozo, Bettina Lado, Ana Inés Moltini, Esteban Vicente, Joanna Lado

**Affiliations:** 1 Instituto Nacional de Investigación Agropecuaria INIA, Camino al Terrible s/n, Salto 50000, Uruguay; pedrozofavierp@gmail.com (P.P.); amoltini@inia.org.uy (A.I.M.); evicente@inia.org.uy (E.V.); 2 Departamento de Biometría, Estadística y Computación, Facultad de Agronomía, Universidad de la República, Sayago 780, Montevideo 12900, Uruguay; blado@fagro.edu.uy

**Keywords:** fruit quality, climate change, strawberry, quality stability, environmental conditions

## Abstract

Strawberry, a profitable crop, adapts well to diverse environments. This study evaluated the stability of fruit quality in different strawberry varieties produced as green plants without chilling during the harvest season in northern Uruguay. The focus was on understanding the impact of harvest date versus agricultural practices (different orchards and growers) on key fruit quality variables such as color, firmness, sugars, and acidity. Results indicated that the INIA Yrupé variety showed greater stability in external coloration and sugar content across harvest dates, suggesting that standardizing fruit coloration is viable under challenging conditions. INIA Guapa consistently met minimum requirements for soluble solids and ratio, with stable acidity and red coloration. Firmness was positively correlated with ratio (r = 0.81) and negatively impacted by rainfall 2–3 days before harvest. The findings suggest that harvest date has a more significant impact on INIA Yrupé fruit quality than growing conditions or practices, with variability observed in firmness and ratio but minimal variation in fruit coloration. These insights highlight the importance of harvest timing for maintaining stable fruit quality traits, which is crucial for breeding programs and ensuring consistent sensory quality and crop profitability.

## 1. Introduction

Strawberry (*Fragaria × ananassa* Duch.) is one of the most essential economical fruit crops, widely adapted to numerous and diverse growing conditions. Its appealing flavor and aroma attract consumers’ interest, thus establishing its wide acceptance as a fruit of choice [1,2]. Strawberry fruit ripening results in the accumulation of multiple sugars and a decrease in organic acids, alongside the accumulation of anthocyanins and volatiles [3,4,5] that contribute to typical and appreciated flavor. This process unfolds in approximately 8 to 10 days, a crucial period where many processes are triggered and where the influence of agricultural management practices and environmental factors could directly have an impact on fruit quality [6,7,8,9].

During strawberry maturation, there is a pronounced increase in total soluble sugars, including glucose, fructose, and sucrose, particularly from the green to red stages [10]. The principal organic acid in strawberries is citrate, whose concentration decreases during fruit maturation. Anthocyanins, constituting the most abundant phenolic compounds in strawberries, play a crucial role in conferring the typical coloration of the fruit and accumulate during the ripening process [11,12]. The predominant anthocyanins in strawberries are pelargonidin-3-glucoside, accounting for 89–95% of the total anthocyanin content, and cyanidin 3-glucoside, which comprises 3.9–10.6% [13,14]. Some studies suggest a higher anthocyanin content in smaller fruit but an opposite trend for total sugars, with the genotype exerting a significant influence [15]. Apart from serving as potent antioxidants, these compounds contribute to the visual appearance of this fruit, influencing consumer appreciation of this berry [16]. In contrast, non-anthocyanin phenolics represent the most abundant antioxidants in green fruit, typically undergoing a decrease during fruit ripening [12].

Fruit quality has predominantly been investigated in genotypes and plants subjected to pre-planting chilling exposure. This exposure occurs naturally in nurseries or artificially supplemented, as exemplified in California conditions [17]. The chilling process entails inducing the plant’s reproductive stage through exposure to low temperatures (0–7 °C), with specific requirements among cultivars [18]. Low temperatures facilitate the accumulation of reserves in crowns and roots, enhancing plant quality after sprouting. That results in improved plant vigor, early production, fruit size, and increased yield, ultimately influencing production patterns [19]. This pre-conditioning process contributes to developing more robust plant vigor and large, well-shaped, and high-quality fruits [20].

Climate change is significantly impacting agriculture and influencing planting decisions. Global warming, particularly in temperate zones, may shift towards production in warmer areas, necessitating the identification of genotypes with low to null chilling requirements [21]. Elevated temperatures are anticipated to result in smaller fruit size and diminished sugar accumulation, compromising strawberries’ marketability and profitability in subtropical regions. However, the success of cultivars adapted to warmer conditions is crucial for sustaining or expanding strawberry production globally [22]. One major challenge in achieving this objective lies in maintaining fruit quality. Non-chilled plants tend to exhibit more significant variability during the productive season, limiting their cultivation, especially in tropical or subtropical regions [22]. Given this uncertain scenario, breeding programs should prioritize the development of strawberry varieties tailored for those zones, particularly in high-temperature and rainy, humid environments. These genotypes should demonstrate resilience to temperature fluctuations, possess the ability to thrive with minimal chilling, initiate flower buds in most environments, and retain desired organoleptic and nutritional properties [21,22,23].

Under the climatic conditions prevailing in North Uruguay, characterized by subtropical weather, the strawberry harvest season extends for a minimum of 6 months, encompassing approximately 40 to 60 harvests. The primary productive systems are based on non-chilled or fresh plants cultivated in greenhouses, macro-tunnels, and micro-tunnels, facilitating production during the autumn, winter, and early spring [24]. These structures serve to mitigate extreme temperatures, provide more diffuse and uniform illumination, and offer protection against heavy rains and frosts during the winter and early spring [25]. Tunnels and greenhouses also confer the advantage of yielding high-value early fruit during the off-season (late autumn and winter), enabling production in colder weather [25]. However, under such a productive system, fruit quality varies drastically, depending on environmental conditions and/or agricultural practices [6,7,8,9]. Studies focusing on the sensory characteristics of different strawberry genotypes throughout a 1-year harvest season revealed noteworthy differences, underscoring the pivotal influence of the harvest date [26,27].

Uruguay needs to gain altitude or latitude for the production of frigo plants. Given the absence of cold exposure before planting, these plants exhibit fewer reserves in the crown, reduced vegetative development, and rapid fructification to provide earlier fruit with higher market value (precocity). A challenge in this approach lies in the considerable intra- and inter-annual variation in weather conditions during the strawberry cycle [28], which typically impacts fruit yield and quality during ripening [29]. This effect could be more pronounced in fresh plants without adequate reserves to sustain or fulfill fructification needs. Therefore, it is of great interest to explore the contribution of breeding to ameliorate or modulate fruit quality changes associated with environmental factors, particularly under a productive model based on fresh plants. These plant production systems ensure fruit availability and quality, and concerns related to global temperature rise, where frigo plants would be more expensive or scarce.

This study aimed to evaluate the stability of fruit quality attributes—including external and internal color, firmness, soluble solids (SS), sugar content, titratable acidity (TA), anthocyanins, and phenolic compounds—in different strawberry varieties cultivated as fresh plants during the harvest season, at three distinct time points, in northern Uruguay. Additionally, we sought to elucidate the relative influence of the harvest date vs. agricultural practices (different orchards and growers) on key and simple fruit quality traits (color, firmness, SS and TA) in a leader variety. Fruit quality reproducibility is also a condition for marketing, differentiation, and valorization of new products.

## 2. Materials and Methods

### 2.1. Field Experiments and Fruit Harvest

#### 2.1.1. Genotypes Behavior among Harvest Dates

In the 1st year of experiments (2019), six different strawberry genotypes from INIA’s breeding program (‘INIA Guapa’, ‘INIA Ágata’, ‘INIA Yrupé’, ‘Q67.3’, ‘T17.4’, and ‘U20.4’) were cultivated in a greenhouse situated in a commercial orchard located in Colonia 18 de Julio (Salto, Uruguay). The experimental design was a split plot with three replicates per treatment, where genotype was considered as the main plot and harvest date as a split-plot factor. The experimental unit (each plot) consisted of 30 plants. The harvest index was defined based on a red coloration in 80–100% of the fruit surface. Thirty fruits from each genotype (three replicates of 10 fruits) were harvested on three specific dates: 5th of June, 8th of August, and 25th of September.

After harvest, fruits were promptly transported to the laboratory and subjected to the following determinations. Ten fruits per replicate were frozen in liquid nitrogen, ground into a fine powder, and stored at −30 °C for further analysis.

#### 2.1.2. Fruit Quality Stability in INIA Yrupé

INIA Yrupé was selected for a 2nd-year study (2021) due to its significance in the region and its demonstrated stability in fruit quality, particularly in sugars, observed in the preceding harvest season. Fully ripe fruit was harvested at five different moments throughout the harvest season: 30th of June, 29th of July, 24th of August, 22nd of September, and 25th of October. This study encompassed five contrasting growing conditions, incorporating protective structures and various agricultural practices. All growers, with a long-standing history as traditional strawberry producers in Salto, Uruguay, were identified numerally from 1 to 5: 1. greenhouse in Colonia 18 de Julio, 2. macro-tunnel in Colonia 18 de Julio, 3. macro-tunnel in Colonia Gestido, 4. micro-tunnel Colonia Gestido, and 5. micro-tunnel in Parada Viña. From each location/grower, four samples of 30 fruits each were harvested for analysis.

### 2.2. Physicochemical Analyses and Free Sugars Determination

Three and four replicates, each consisting of 10 fruits per genotype, were employed for the genotypes and INIA Yrupé experiments, respectively. Internal and external colors were evaluated by determining the CIELAB coordinates (L*, a*, and b*) using a Minolta CR-400 colorimeter, with three measurements taken per fruit. The redness of the fruit was estimated using a reference color index (CI), calculated using the formula CI = 2000 − a*/L* × (a^2^ + b^2^)^0.5^, a metric developed for tomato fruit, where higher values indicate more intense red coloration [30]. Fruit firmness was assessed as the maximum penetration force, measured in Newtons (N), required to penetrate 3 mm into the tissue, utilizing a 6 mm diameter cylindrical probe on a TA-XTplus texture analyzer (Stable Micro Systems). For strawberry juice extraction, 10 fruits per replicate were processed in a Philips domestic juicer. TA and SS content were determined using the method described by Lado et al. [31].

To analyze free sugars (fructose, glucose, sucrose, and total sugars), the method outlined by Li et al. was adjusted [32]. Briefly, 0.5 g of frozen ground tissue (−80 °C) was homogenized in 5 mL of ethanol (80:20, *v*/*v*) (Merck, Darmstadt, Germany), with the addition of 100 µL of fucose (Sigma Aldrich, St. Louis, MO, USA) as an internal standard (0.03 g/mL). The mixture underwent sonication at 30 °C for 45 min and centrifugation at 7000 rpm and 20 °C for 15 min. The supernatant was transferred to a new tube, and ethanol was removed by speed-vac evaporation. The dried residue was dissolved in 0.5 mL of milli Q water and filtered through 0.45 µm PDVF filters (Merck Millipore, Darmstadt, Germany).

Separation and quantification of glucose, fructose, and sucrose were conducted using Agilent Technologies-1260 Infinity II high-performance liquid chromatography (HPLC) with an Agilent ZORBAX Carbohydrate column (Agilent, ASM, Montevideo, Uruguay). An acetonitrile/water milli Q solution (75:25, *v*/*v*) served as the mobile phase in isocratic conditions with a 2.0 mL/min flow rate. The concentration of analyzed sugars was calculated by comparing peak areas with those obtained from solutions of fructose, glucose, and sucrose (Sigma Aldrich, St. Louis, MO, USA) of known concentrations. Results were adjusted based on fucose recovery, and the outcomes were expressed as g in 100 g of fresh weight (FW).

### 2.3. Bioactive Compounds Anthocyanins and Phenolics

The concentration of anthocyanins and total phenolics was determined from a methanolic extract obtained following the procedure outlined in Ferrari et al. [33]. Five milliliters of an 80:20 methanol/water mixture (Merck, Darmstadt, Germany) was added to 1 g of frozen and grounded strawberry tissue. The mixture was vigorously shaken for 1 min, sonicated for 6 min at room temperature, and then centrifuged at 5000 rpm and 4 °C for 20 min. The anthocyanins content was determined as follows: a 500 µL aliquot of the extract was diluted with 4500 µL of potassium chloride or sodium acetate buffers (Merck, Germany). The absorbance was measured at the maximum absorption wavelength (495 nm) and 720 nm using an Evolution 260 BIO spectrophotometer, Thermo Fischer Scientific (Waltham, MA, USA). The absorbance (A) of anthocyanins was calculated as follows: A = (A_495_ − A_700_)_pH 1.0_ − (A_495_ − A_700_)_pH 4.5_, and the concentration of anthocyanins was calculated using the extinction coefficient of cyanidin 3-glucoside. Results were expressed as mg cyanidin 3-glucoside equivalents (CE) in 100 g of fresh weight (FW) [26].

For total phenolic determination, the method described by Sánchez-Rangel et al. was employed [34] with some modifications. In brief, 300 µL of the methanolic extract (diluted 1:10) was mixed with 1.5 mL of Folin–Ciocalteau reagent (diluted 1:10) (Merck, Darmstadt, Germany). The mixture was vigorously shaken and allowed to stand for 2 min. After adding 1.2 mL of 7% sodium carbonate (Merck, Darmstadt, Germany), it was allowed to stand for 2 h in the dark. Methanol 80:20 was used as a blank. The absorbance was determined at a wavelength of 760 nm (Evolution 260 BIO, Thermo Fischer Scientific, Waltham, MA, USA). A calibration curve was constructed using gallic acid (Merck, Darmstadt, Germany) as a reference. Results were expressed as mg of gallic acid equivalents (GAE) in 100 g of fresh weight (FW).

### 2.4. Statistical Analysis

To compare the behavior of different genotypes across various harvest dates, the variables external color, soluble solids, firmness, individual sugars, and total anthocyanins were analyzed using analysis of variance (ANOVA) with a factorial design of three harvest dates and six genotypes. The main plot was considered genotype, while the split-plot design with two factors was represented by harvest date. Statistical analyses were performed using the open-source software R. Treatment means were compared using Tukey’s Test. Additionally, a mean comparison of physicochemical fruit characterization data for different genotypes at each harvest date was performed (Supplementary Table S1).

For the evaluation of INIA Yrupé, the relationship between variables (firmness, SS, TA, ratio, individual sugars, anthocyanins, and phenols) and their responses across different harvest dates under contrasting growing conditions (1 to 5) was investigated using Principal Component Analysis (PCA) and Pearson Correlation Analysis. PCA was conducted using the FactoMineR and factoextra packages in the R software (https://www.R-project.org/) environment, considering standardized data.

## 3. Results

The results regarding external coloration indicated that INIA Guapa, INIA Yrupé, and Q67.3 exhibited lower variability in CI among harvests. Higher values were observed for U20.4 in September (54.5), followed by June (45.9) and August (42.3) (Table 1). Similarly, INIA Ágata displayed a more intense red coloration in later harvest, with no differences between June and August, positioning it as one of the most reddish genotypes in both June (50.9) and September (56.4) harvests (see Supplementary Table S1).

There were significant differences among genotypes in internal coloration, with U20.4 standing out due to its intense red internal color at early- and mid-harvests (26.2 and 25.3, respectively), whereas the rest of the genotypes (except for T17.4) exhibited the opposite behavior, with higher values observed in September (ranging from 21 to 27). Correlation analysis revealed that external and internal fruit coloration were not significantly correlated (*p* > 0.05; see Supplementary Figure S1).

Fruit firmness values, as shown in Table 1, were generally higher in September for all genotypes (ranging from 5.7 to 7.4 N), except for T17.4, which exhibited more stable but lower values compared to other genotypes (ranging from 3.0 to 3.3 N). U20.4 demonstrated higher firmness values than the other genotypes in June and August, with good stability observed in early harvests (see Supplementary Table S1). In contrast, INIA Guapa displayed significant variations in fruit firmness, with particularly high values in September (7.4 N) compared to fruit harvested earlier (2.4–2.8 N) (Table 1).

Analysis incorporating data from all genotypes revealed a significant positive correlation between internal or external coloration and fruit firmness (0.59 and 0.39, respectively). Fruit firmness also exhibited a positive correlation with the fruit ratio (0.31) (refer to Supplementary Figure S1). The relationship between SS and TA followed a consistent but opposite pattern. Early harvest, occurring in June, generally exhibited lower SS levels (ranging from 6.4 to 7.2°Brix), coupled with higher acidity levels (ranging from 0.47 to 0.72%). Conversely, a significant increase in SS was observed during the September harvest, with values ranging from 7.0 to 9.6°Brix, coinciding with a decrease in acidity, which ranged from 0.39 to 0.56% during this period. Consequently, the fruit ratio consistently peaked in September (ranging from 15.4 in INIA Ágata to 22.8 in INIA Guapa) across all genotypes, while it was lower during the June harvest (ranging from 9.4 in INIA Ágata to 15.3 in INIA Guapa).

Noteworthy observations include minimal variations in SS content but a decrease in TA throughout the harvest season for Q67.3 (SS ranging from 6.9 to 7.3°Brix) and U20.4 (SS ranging from 6.4 to 6.9°Brix) (see Table 1). INIA Guapa demonstrated remarkable stability in acidity (ranging from 0.42 to 0.47%) throughout the harvest season while exhibiting extreme variability in SS (ranging from 7.2 to 6.9°Brix). Similarly, INIA Yrupé displayed higher SS levels during the August harvest (8.59°Brix) and lower SS levels during June (6.48°Brix).

**Figure 1:**
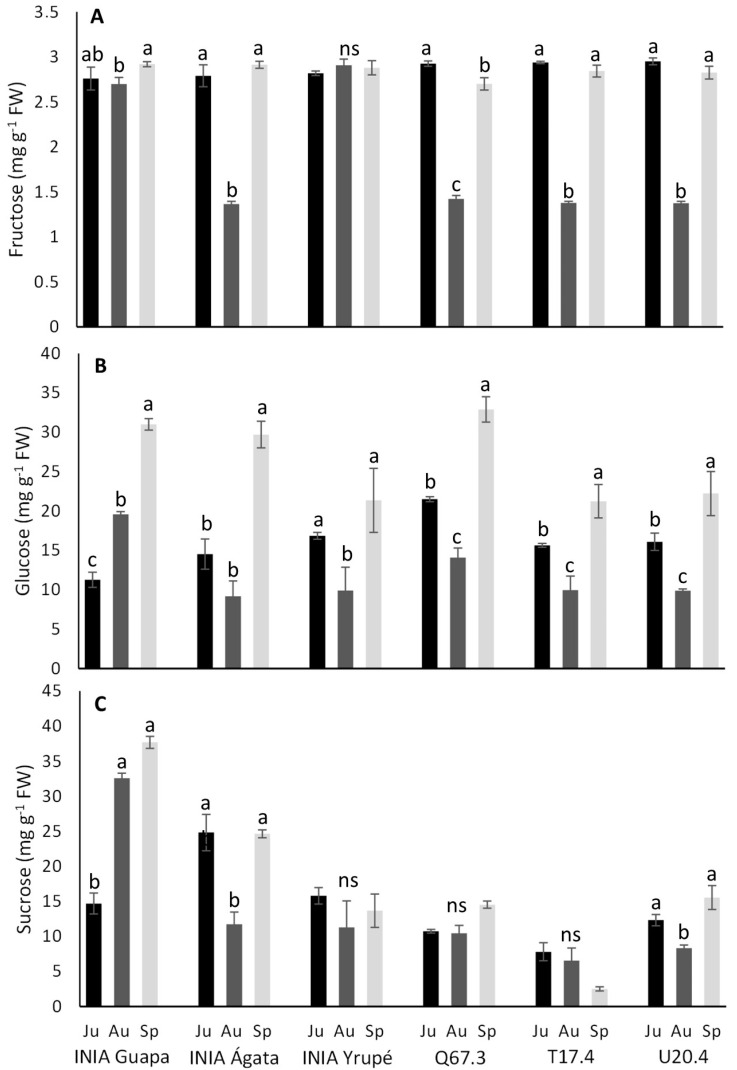
Individual sugar concentrations of glucose (**A**), fructose (**B**), and sucrose (**C**) (mg g^−1^ FW) for six different genotypes and three harvest dates (June [Ju], August [Au], and September [Sp]). Means followed by the same letter within each genotype across dates do not differ significantly (*p* < 0.05, Tukey).

When examining individual sugars (Figure 1), fructose emerged as the least abundant compound across all genotypes, with concentrations ranging from 1.4 to 3.0 mg g^−1^ FW, followed by sucrose (2.5–38 mg g^−1^ FW) and glucose (9.0–33 mg g^−1^ FW). Fructose levels exhibited minimal changes among harvest dates, being lower in August for many genotypes (INIA Ágata, Q67.3, T17.4, and U20.4) while remaining unchanged in INIA Yrupé or showing little fluctuations in INIA Guapa (Figure 1A). Glucose content (Figure 1B) was notably higher during the September harvest (ranging from 21.2 to 32.9 mg g^−1^) for almost all the genotypes (showing a similar trend to SS), with lower values observed in August (9.1 to 19.6 mg g^−1^) and June (11.3 to 21.5 mg g^−1^). Furthermore, INIA Yrupé exhibited stability in glucose content with no significant differences between early and late harvest. Regarding sucrose (Figure 1C), INIA Guapa displayed a sharp rise in August and September, registering very high values (32.6 mg g^−1^ and 37.7 mg g^−1^, respectively), at least threefold higher than the other genotypes. INIA Ágata also reached its peak sucrose content (nearly 25 mg g^−1^) in June and September (Figure 1C). INIA Yrupé showed no significant changes in sucrose content throughout harvest season (averaging 13.6 mg g^−1^), mirroring the patterns observed in Q67.3 (11.9 mg g^−1^) and T17.4 (5.6 mg g^−1^), while U20.4 exhibited slightly lower values in August (8.3 mg g^−1^) compared to June (12.3 mg g^−1^) and September (15.5 mg g^−1^). A positive correlation was observed between soluble solids and the individual sugars glucose (0.41) and sucrose (0.47).

**Figure 2:**
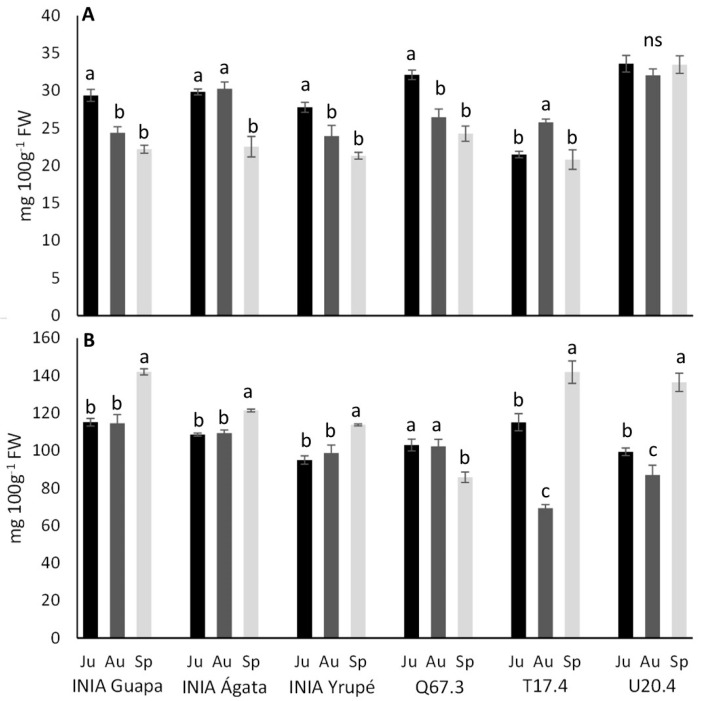
Total anthocyanins (**A**) and total phenolics (**B**) concentrations (mg 100 g^−1^ FW) for six different genotypes and three harvest dates (June [Ju], August [Au], and September [Sp]). Means followed by the same letter within each genotype across dates do not differ significantly (*p* < 0.05, Tukey).

Anthocyanins content (Figure 2A) exhibited variations among harvest, with the exception of U20.4, which consistently displayed the highest content (32–34 mg 100 g^−1^) of these compounds and maintained stability throughout the harvest season. Conversely, most genotypes experienced a decline in total anthocyanin content in later harvests (August: 24–30 mg 100 g^−1^ and September: 21–24 mg 100 g^−1^), except for T17.4 and INIA Ágata. Notably, no correlation was observed between internal or external coloration and anthocyanin content (see Supplementary Figure S1). Total phenolics content (Figure 2B) remained relatively stable in INIA Guapa (115–142 mg 100 g^−1^), INIA Ágata (108–121 mg 100 g^−1^), and INIA Yrupé (95–114 mg 100 g^−1^), with a slight increase noted in September (14%) compared to earlier harvests. Conversely, Q67.3 exhibited slightly lower values (15%) in September, while T17.4 and U20.4 experienced a transient decrease in total phenolics content in August, followed by an increase to 142 and 136 mg 100 g^−1^ in September, respectively. Total phenolics showed a positive correlation with soluble and individual sugars (0.36–0.49), whereas anthocyanins exhibited a negative correlation with glucose (−0.34) and SS (−0.52) (Supplementary Figure S1).

**Figure 3:**
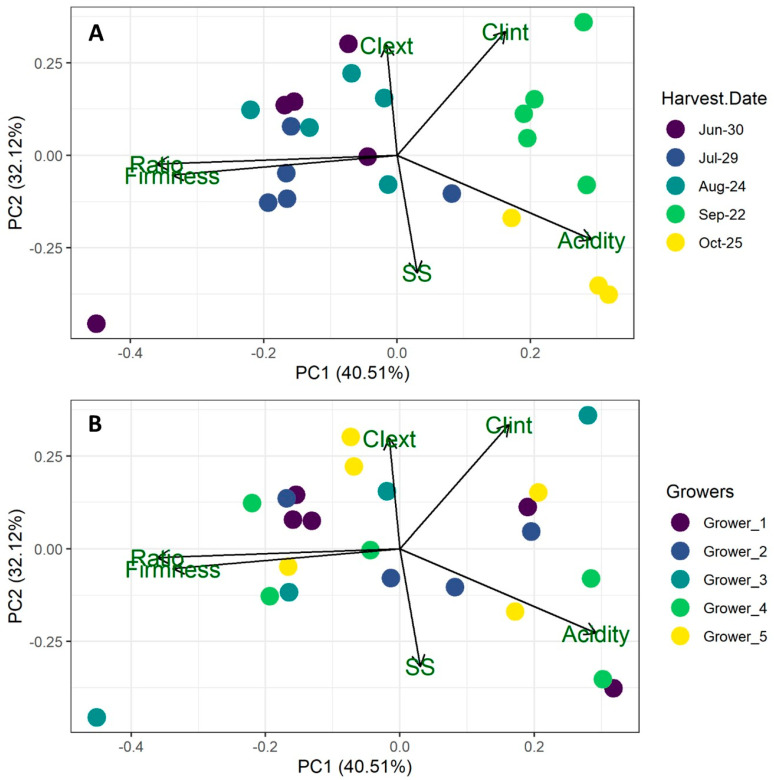
Biplot of Principal Component Analysis (PCA) of the matrix of means for six quality variables (arrows): external color index (CIext), internal color index (CIint), soluble solids (SS), firmness, titratable acidity (Acidity), and the ratio between them. This biplot represents each grower and harvest date (points) to visualize the relationships between variables, genotypes, and growers by reducing the dimensionality of the data. The points are colored by harvest date (**A**) and growers (**B**).

INIA Yrupé was selected for further investigation due to its significance as one of the predominant and widely cultivated varieties in the region. This study encompassed five distinct harvest moments and five contrasting growing conditions involving various management practices and protective structures. Fully ripe fruit was harvested from five different growers (1 to 5) in June, July, August, September, and October. Easily measurable quality parameters, including color, firmness, SS, TA, and ratio, were selected for analysis, and the results were depicted in a PCA chart (Figure 3). The first two components of the PCA accounted for 72.63% of the total variability: Principal Component (PC) 1 (40.51%), primarily associated with firmness, ratio, and TA; and PC2 (32.12%), linked to color and SS. The samples were differentiated along PC1 by harvest date, predominantly influenced by firmness, ratio, and TA, while there was no distinct separation or aggregation of samples based on growing conditions (Figure 3). Notably, INIA Yrupé samples harvested in October and September exhibited higher acidity and lower firmness and ratio. The latter two variables showed a strong positive correlation (0.81; Supplementary Figure S2).

## 4. Discussion

Results revealed significant intra-annual variability in fruit quality across nearly all genotypes, influenced by harvest date and, thereby, the environmental conditions during fruit ripening. However, the extent of this variability varied depending on the genotype, with some displaying greater fluctuations than others, contingent upon the analyzed variables. This aligns with findings from similar studies on other varieties and agricultural contexts [8]. Authors like Cervantes et al. [26] introduced the concept of plasticity or plastic behavior to describe varieties more responsive to environment cues, exhibiting greater shifts in fruit quality (e.g., T17.4 or U20.4), contrasting with more stable genotypes like INIA Guapa, particularly in color and acidity, and INIA Yrupé in color and individual sugars. These stable genotypes offer the assurance of more consistent fruit quality. The changes in fruit composition may be linked to adaptability to dynamic environments, which modify fruit quality in response to various challenges. However, such adaptative responses may be less relevant for genotype selection when aiming to standardize fruit quality during a prolonged and variable harvest season [9].

Despite the variance in SS, INIA Yrupé demonstrated a more stable glucose, fructose, and sucrose content among harvest dates compared to other genotypes. This seeming contradiction could be attributed to the influence of other compounds, like organic acids or amino acids, on SS oscillation [26,35]. Moreover, the former could also underlie the moderate (but positive) correlation between glucose (0.41) and sucrose (0.47) with total SS (measured as °Brix) (Supplementary Figure S1). Correlation analysis also suggests that sugar levels could positively influence total phenolic accumulation in strawberries but negatively impact anthocyanin content, revealing a tight connection between these metabolites. Ratio and firmness were positively correlated (0.81), with higher values observed in September and lower values in the early June harvest. Both variables are crucial, as firmness directly influences the postharvest life of this perishable fruit, while a higher ratio is associated with better consumer liking scores [27,36,37]. Therefore, fruit harvested in September from these genotypes tends to be more valuable from a fresh market perspective, with an expected higher overall consumer liking [16].

A thorough investigation was undertaken on INIA Yrupé, focusing on its consistent fruit quality attributes related to CI and sugars, coupled with its extensive cultivation coverage, encompassing nearly 80% of the strawberry cultivation area in northern Uruguay. This study included an examination of five different harvest dates and growers operating under diverse growing conditions, with specific attention devoted to key physicochemical parameters easily measurable in the field. PCA results delineated a distinct segregation of strawberry samples based on harvest date, predominantly associated with the ratio, acidity, and firmness (PC1). Notably, no discernible separation based on growing conditions or growers was observed. Later harvests exhibited heightened acidity and internal color but diminished ratio and firmness compared to earlier ones (Figure 3). The findings underscored the significant impact of harvest date on fruit quality, overshadowing the variability attributed to growing practices or agricultural management techniques. Furthermore, clustering of samples based on harvest date was evident in the PCA, while no discernible patterns could be observed among growers, irrespective of their environmental productive systems and management practices.

These findings highlight the paramount influence of harvest timing on fruit quality for INIA Yrupé within this specific agricultural framework, outweighing the impact of growing practices. Reduced sweetness and firmness are anticipated following prolonged cloudy periods with reduced radiation, high humidity, and long thermal amplitude, irrespective of the employed productive system or management practices [9,26]. The choice of plant type in the region partially elucidates the significant variation in fruit quality and its pronounced environmental dependency. Frigo plants utilizing stored energy for sprouting and fruiting ensure greater stability throughout the harvest cycle [38]. Conversely, fresh plants produce fewer but larger fruits over an extended harvesting period [38], lacking reserves in their roots and exerting minimal influence on fruit quality. Consequently, the selection of suitable varieties aligned with grower objectives becomes increasingly pertinent under such circumstances.

Data gleaned from this 2-year study on INIA Yrupé suggested potential discrepancies in fruit quality between years, warranting further exploration. Analogous results were observed in different years under subtropical conditions [39]. For instance, fruit firmness exhibited variability, being higher in September during the 1st year but lower in both September and October in the subsequent year. Similarly, TA and ratio also showcased disparate trends in both years, with September exhibiting lower and higher values, respectively. These variations could be linked to environmental factors in the 8 to 10 days preceding harvest each year (Supplementary Tables S2 and S3), critical for the strawberry ripening process and metabolite alterations [8,40,41]. Uruguay’s substantial inter-annual weather variability [28], compounded by climatic shifts over the past decade [42], directly influences fruit firmness via temperature, rainfall and relative humidity fluctuations [8]. During genotype comparison experiments, leading up to the June harvest, the weather was characterized by persistent rainfall, overcast skies, and high humidity during 6 out of the 10 days preceding harvest. The total rainfall measured 6.7 mm, with only 4 h of daily sunlight exposure and low radiation levels (193 cal cm^2^ d), coupled with high relative humidity (88%) (Supplementary Table S2). Conversely, in the INIA Yrupé experiment (Supplementary Table S3), total rainfall surged to 83 mm, primarily concentrated within a single day, occurring 5 days before the harvest. These disparities in rainfall distribution within the 10-day period before harvest may account for the discrepancies in fruit firmness observed during the June harvest in the 1st and 2nd years, respectively. Prior research conducted in subtropical regions has established a correlation between pre-harvest rainfall and reduced fruit firmness, as well as adverse effects on fruit coloration [39].

Similarly, preceding the September harvest, the weather exhibited sunny conditions with negligible rainfall (totaling 0 mm), accompanied by increased radiation (442 cal cm^2^ d) and 8.4 h of sunlight exposure (heliophany). Significantly, there were notable variations between maximum and minimum temperatures, indicative of an average thermal amplitude of 14.5 °C. These favorable weather conditions contributed to firmer fruit during the later harvest date that year. Conversely, during INIA Yrupé evaluation, accumulated rainfall over the preceding 10 days amounted to 5.7 mm, occurring 2 to 3 days before harvest, resulting in diminished fruit firmness.

The findings of this study indicate potential trends that suggest correlations between specific environmental factors and quality traits. However, no statistically significant correlations were identified (*p*-value ≤ 0.05) during this 2-year study period (see Supplementary Figure S3), which may be attributed to limitations inherent in the dataset. Moreover, it is crucial to acknowledge that environmental conditions exhibit considerable inter-annual variability, which may render conclusions derived from data collected over just 2 years potentially unreliable for extrapolation to future scenarios. Nonetheless, the observed trends—such as the relationship between firmness and the ratio relative to thermal amplitude—provide a valuable basis for further investigation and replication to better account for natural variability. A more rigorous approach would involve the implementation of multi-year trials to thoroughly evaluate the influence of environmental conditions on the traits of interest.

INIA Yrupé demonstrates remarkable stability in fruit coloration across different harvest times and growers, indicating its potential as a consistent option for standardizing fruit coloration, even under different growing conditions. Given the crucial role of color in consumer acceptance of strawberries [16,43], this consistency enhances the variety’s market value, despite color being a higher variable trait among different varieties and/or weather conditions [8,12,44]. Regarding internal quality, INIA Guapa was the only genotype that consistently meets the minimum threshold of 7°Brix [45] regardless of the harvest date, whereas commercially available genotypes like INIA Yrupé and INIA Ágata exceed 7°Brix in August and September but fall short in June. Notably, all the genotypes exhibited lower TA levels than the maximum recommended (0.8%) for acceptable flavor [45].

## 5. Conclusions

INIA Yrupé emerged as the most consistent genotype across various harvest dates concerning fruit color and individual sugar content, while clones Q67.3 and U20.4 demonstrated higher stability regarding soluble solids. Notably, INIA Guapa consistently maintained color and acidity levels, ensuring it consistently met the minimum required soluble solids and exhibited a higher ratio, irrespective of the harvest date. U20.4 distinguished itself with intense internal coloration and superior firmness. Depending on market objectives, these varieties may exhibit varying degrees of stability during the harvest season under a non-chilled plant-based productive system. This information could be useful not only for breeding purposes but also aid growers in selecting new materials suited for planning future global warming scenarios.

This study’s results indicate that, within this productive system and for INIA Yrupé specifically, different harvest times exert a more significant influence on fruit quality (color, firmness, soluble solids, acidity, and ratio) compared to varying management conditions among growers. A linkage was observed between rainfall 10 days before harvest and fruit firmness, with this variable positively associated with fruit ratio (r = 0.81). Later harvests consistently exhibit higher acidity and internal color but a lower ratio and firmness than earlier ones, regardless of growing conditions. Additionally, inklings of a substantial inter-annual variability were observed, particularly in firmness and ratio variables, underscoring the significant environmental influence on certain quality parameters within such productive systems. INIA Yrupé demonstrated exceptional stability in fruit coloration among different harvest times and growers, suggesting its potential as a reliable option for standardizing fruit coloration even under challenging environmental conditions.

## Figures and Tables

**Table 1:** Fruit quality (external and internal color index), firmness, acidity, soluble solids, and ratio for six different genotypes and three harvest dates (June, August, and September).

	INIA Guapa	INIA Ágata	INIA Yrupé	Q67.3	T17.4	U20.4
Harvest date	**Color Index (external)**					
Jun	45.1 ± 0.36 ^a^	50.9 ± 0.87 ^b^	45.2 ± 0.52 ^a^	47.5 ± 0.58 ^a^	47.2 ± 0.34 ^ab^	45.94 ± 0.19 ^b^
Aug	44.8 ± 0.37 ^a^	48.5 ± 1.22 ^b^	46.6 ± 0.82 ^a^	48.2 ± 1.42 ^a^	48.3± 1.27 ^a^	42.28 ± 0.72 ^c^
Sept	46.8 ± 0.56 ^a^	56.4 ± 0.29 ^a^	46.5 ± 1.05 ^a^	46.7 ± 1.79 ^a^	45.2 ± 0.92 ^b^	54.5 ± 0.57 ^a^
	**Color Index (internal)**					
Jun	18.2 ± 0.26 ^b^	14.2 ± 0.14 ^b^	17.1 ± 0.81 ^b^	15.7 ± 0.96 ^b^	14.5 ± 0.38 ^b^	26.2 ± 0.51 ^a^
Aug	21.0 ± 0.69 ^a^	21.0 ± 0.46 ^a^	18.4 ± 0.55 ^b^	19.5 ± 0.56 ^a^	18.9 ± 0.13 ^a^	25.3 ± 0.73 ^ab^
Sept	21.2 ± 0.51 ^a^	22.4 ± 0.65 ^a^	27.2 ± 0.55 ^a^	20.9 ± 0.35 ^a^	13.6 ± 0.58 ^b^	23.9 ± 0.40 ^b^
	**Firmness (N)**					
Jun	2.44 ± 0.04 ^b^	4.85 ± 0.15 ^b^	4.80 ± 0.40 ^ab^	3.46 ± 0.18 ^b^	3.09 ± 0.17 ^a^	5.63 ± 0.30 ^b^
Aug	2.76 ± 0.08 ^b^	4.25 ± 0.22 ^b^	4.16 ± 0.26 ^b^	3.98 ± 0.05 ^b^	3.34 ± 0.20 ^a^	5.05 ± 0.04 ^b^
Sept	7.38 ± 0.30 ^a^	6.16 ± 0.46 ^a^	5.69 ± 0.29 ^a^	5.89 ± 0.19 ^a^	3.06 ± 0.22 ^a^	7.35 ± 0.45 ^a^
	**Titratable Acidity**					
Jun	0.47 ± 0.01 ^a^	0.72 ± 0.03 ^a^	0.54 ± 0.01 ^a^	0.65 ± 0.03 ^a^	0.51 ± 0.07 ^ab^	0.55 ± 0.01 ^a^
Aug	0.46 ± 0.01 ^a^	0.57 ± 0.02 ^b^	0.54 ± 0.01 ^a^	0.48 ± 0.01 ^b^	0.43 ± 0.03 ^b^	0.47 ± 0.02 ^ab^
Sept	0.42 ± 0.01 ^a^	0.56 ± 0.03 ^b^	0.39 ± 0.01 ^b^	0.43 ± 0.02 ^b^	0.54 ± 0.00 ^a^	0.39 ± 0.00 ^b^
	**Soluble Solids**					
Jun	7.18 ± 0.15 ^c^	6.72 ± 0.17 ^b^	6.48 ± 0.04 ^c^	6.87 ± 0.03 ^a^	5.75 ± 0.14 ^c^	6.39 ± 0.27 ^a^
Aug	8.59 ± 0.12 ^b^	7.16 ± 0.03 ^b^	8.59 ± 0.12 ^a^	7.20 ± 0.23 ^a^	6.79 ± 0.29 ^b^	6.42 ± 0.01 ^a^
Sept	9.61 ± 0.07 ^a^	8.64 ± 0.36 ^a^	7.44 ± 0.30 ^b^	7.34 ± 0.07 ^a^	8.93 ± 0.06 ^a^	6.95 ± 0.24 ^a^
	**Ratio**					
Jun	15.3 ± 0.63 ^c^	9.37 ± 0.39 ^c^	11.9 ± 0.21 ^c^	10.6 ± 0.51 ^c^	11.8 ± 1.70 ^c^	11.6 ± 0.63 ^c^
Aug	18.7 ± 0.36 ^b^	12.6 ± 0.43 ^b^	16.0 ± 0.35 ^b^	14.9 ± 0.34 ^b^	16.0 ± 1.51 ^b^	13.6 ± 0.45 ^b^
Sept	22.8 ± 0.71 ^a^	15.4 ± 0.74 ^a^	19.0 ± 0.78 ^a^	17.1 ± 1.05 ^a^	16.5 ± 0.12 ^a^	17.7 ± 0.65 ^a^

Means (±SE) followed by the same letter within each genotype across harvest dates do not differ significantly (*p* < 0.05).

## Data Availability

Data are contained within the article.

## Supplementary Materials

The following supporting information can be downloaded at: https://www.mdpi.com/article/10.3390/plants13213052/s1, Table S1. Fruit quality (external and internal color index), firmness, acidity, soluble solids and ratio for six different genotypes and three harvest dates (June, August and September). Table S2. Climatic data for the 10 days before harvest date (first year-2019). Table S3. Climatic data for the 10 days before harvest date (second year- 2021 experiments). Figure S1. Matrix of correlations for genotypes behavior study (data from all genotypes and harvest dates). Figure S2. Correlations in quality variables (Ratio, Firmness, Soluble solids-SS, Acidity, external color index- CIext and internal color index- CIint) for INIA Yrupé study. Figure S3. Correlations between quality (Ratio, Firmness, Soluble solids-SS, Acidity-TA, external color index- CIext, internal color index- CIint) and environmental variables (Max, Min and Mean temperature, Heliophany, Rainfall, Relative humidity-RH, Radiation and Thermal amplitude) for INIA Yrupé study.
