# Supplementary material for: Exploration of Strawberry Fruit Quality During Harvest Season Under a Semi-Forcing Culture with Plants Nursed Without Chilling
Source: Plants (Basel). 2024 Oct 31;13(21):3052. doi: 10.3390/plants13213052 (PMC11548627; doi:10.3390/plants13213052)
Supplement: Supplementary file 1 [file plants-13-03052-s001.zip › plants-3214522-supplementary.pdf]

Supplementary material

Table S1. Fruit quality (external and internal color index), firmness, acidity, soluble solids and ratio for six different genotypes and three harvest dates (June, August and September).

	**Color Index (external)**			**Color Index (internal)**			**Firmness (N)**		
Variety/Harvest	Jun	Aug	Sep	Jun	Aug	Sep	Jun	Aug	Sep
INIA Guapa	45.1 ± 0.36^{b}	44.8 ± 0.37^{ab}	46.8 ± 0.56^{b}	18.2 ± 0.26^{b}	21.0 ± 0.69^{b}	21.2 ± 0.51^{c}	2.44 ± 0.04^{d}	2.76 ± 0.08^{c}	7.38 ± 0.30^{a}
INIA Ágata	50.9 ± 0.87^{a}	48.5 ± 1.22^{a}	56.4 ± 0.29^{a}	14.2 ± 0.14^{d}	21.0 ± 0.46^{b}	22.4 ± 0.65^{bc}	4.85 ± 0.15^{b}	4.25 ± 0.22^{b}	6.16 ± 0.46^{ab}
INIA Yrupé	45.2 ± 0.52^{b}	46.6 ± 0.82^{ab}	46.5 ± 1.05^{b}	17.1 ± 0.81^{bc}	18.4 ± 0.55^{b}	27.2 ± 0.55^{a}	4.80 ± 0.40^{b}	4.16 ± 0.26^{b}	5.69 ± 0.29^{b}
Q67.3	47.5 ± 0.58^{b}	48.2 ± 1.42^{a}	46.7 ± 1.79^{b}	15.7 ± 0.96^{bcd}	19.5 ± 0.56^{b}	21.0 ± 0.35^{c}	3.46 ± 0.18^{bc}	3.98 ± 0.05^{b}	5.89 ± 0.19^{ab}
T17.4	47.2 ± 0.34^{b}	48.3 ± 1.27^{a}	45.2 ± 0.92^{b}	14.5 ± 0.38^{cd}	18.9 ± 0.13^{b}	13.6 ± 0.58^{d}	3.09 ± 0.17^{cd}	3.34 ± 0.20^{b}	3.06 ± 0.22^{c}
U20.4	45.9 ± 0.19^{b}	42.3 ± 0.72^{b}	54.5 ± 0.57^{a}	26.2 ± 0.51^{a}	25.3 ± 0.73^{a}	23.9 ± 0.40^{b}	5.63 ± 0.30^{a}	5.05 ± 0.04^{a}	7.35 ± 0.45^{a}
Variety		**Acidity**			**Soluble Solids**			**Ratio**	
INIA Guapa	0.47 ± 0.01^{c}	0.46 ± 0.01^{c}	0.42 ± 0.01^{b}	7.18 ± 0.15^{a}	8.59 ± 0.12^{a}	9.61 ± 0.07^{a}	15.3 ± 0.63^{a}	18.7 ± 0.36^{a}	22.8 ± 0.71^{a}
INIA Ágata	0.72 ± 0.03^{a}	0.57 ± 0.02^{a}	0.56 ± 0.03^{a}	6.72 ± 0.17^{ab}	7.16 ± 0.03^{b}	8.64 ± 0.36^{a}	9.37 ± 0.39^{b}	12.6 ± 0.43^{c}	15.4 ± 0.74^{c}
INIA Yrupé	0.54 ± 0.01^{bc}	0.54 ± 0.00^{ab}	0.39 ± 0.01^{b}	6.48 ± 0.04^{abc}	8.59 ± 0.12^{a}	7.44 ± 0.30^{b}	11.9 ± 0.21^{ab}	16.0 ± 0.35^{ab}	19.0 ± 0.78^{b}
Q67.3	0.65 ± 0.03^{ab}	0.48 ± 0.01^{bc}	0.43 ± 0.02^{b}	6.87 ± 0.03^{ab}	7.20 ± 0.23^{b}	7.34 ± 0.07^{b}	10.6 ± 0.51^{b}	14.9 ± 0.34^{bc}	17.1 ± 1.05^{bc}

T17.4	0.51 ± 0.07^{bc}	0.43 ± 0.03^{c}	0.54 ± 0.00^{a}	5.75 ± 0.14^{c}	6.79 ± 0.29^{b}	8.93 ± 0.06^{a}	11.8 ± 1.70^{ab}	16.0 ± 1.51^{ab}	16.5 ± 0.12^{bc}
U20.4	0.55 ± 0.01 ^{bc}	0.47 ± 0.02^{bc}	0.39 ± 0.00^{b}	6.39 ± 0.27^{bc}	6.42 ± 0.00^{b}	6.95 ± 0.24^{b}	11.6 ± 0.63^{ab}	13.6 ± 0.45^{bc}	17.7 ± 0.65^{bc}

Means (± SE) followed by the same letter among genotypes for each harvest date do not differ significantly ($p<0.05$).

Table S2. Climatic data for the 10 days before harvest date (first year-2019)

Date	Thermal amplitude	Radiation cal/cm2/dia	Mean temperature (°C)	Max T (°C)	Min temperature (°C)	RH (%)	Rainfall (mm)	Heliophany (h)
2019-09-24	21.2	518.6	17.3	27.1	3.9	58	0	10.4
2019-09-23	13.2	445	16.6	23.2	7.3	53	0	8.2
2019-09-22	14.8	513.3	17.1	22.8	4	62	0	10.4
2019-09-21	18.7	510.5	15.7	23	3	63	0	10.4
2019-09-20	12.2	498.4	14.6	22	4.8	48	0	10.1
2019-09-19	11.4	470.2	18.1	23.9	10.1	59	0	9.3
2019-09-18	14.3	508.5	17.1	23.1	7	65	0	10.6
2019-09-17	11.6	291.9	13.2	18.6	5	75	0	3.8
2019-09-16	10.1	230.6	16.6	21	15.2	81	0	1.9
2019-09-15	17	431.5	21.3	29.4	12.4	75	0	8.4
**AVG/SUM**	**14.45**	**441.85**	**16.76**	**23.41**	**7.27**	**63.9**	**0**	**8.35**
2019-08-07	12.1	235.3	17.6	23.3	9.5	87	9.9	4.5
2019-08-06	13.9	353.1	16.7	23.7	8.6	81	0	9.3
2019-08-05	14.2	358.1	12.4	19.4	3.9	70	0	9.6
2019-08-04	15.4	353.5	9.6	16.8	0.1	71	0	9.5
2019-08-03	12.8	338.9	9.2	16.1	-1.6	71	0	9
2019-08-02	9.6	349	10.2	15.8	5.1	73	0	9.5
2019-08-01	8.2	117	13.4	17.1	15	91	3.6	0
2019-07-31	14.9	330.2	15.3	15	6.2	82	1.5	8.9
2019-07-30	13.3	251.6	11.1	18.6	5.3	88	0	5.7
2019-07-29	8.9	304.7	13.1	17.9	9	86	0	8
**AVG/SUM**	**12.33**	**299.14**	**12.86**	**18.37**	**6.11**	**80**	**15**	**7.4**
2019-06-04	11.6	262	14.8	20.1	6.7	80	0	7.4
2019-06-03	14.3	291.4	13.4	21.1	4.4	85	0.4	8.7
2019-06-02	9.9	266.2	15.6	23.9	6	86	0.4	7.5
2019-06-01	8.6	231.9	16	21	11	89	0	5.9
2019-05-31	8.8	131.3	15.6	20.6	10.5	91	0.2	1.3
2019-05-30	4.5	109.8	14.3	19.8	12.8	86	5	0.3
2019-05-29	9.5	223.8	16.5	21.4	10.2	86	0	5.4
2019-05-28	4.4	122.1	15.6	17.9	11.6	97	0.3	0.8
2019-05-27	7.6	140.7	18	22.5	11.1	89	0	1.6
2019-05-26	9.3	150.5	17.6	20.3	10.6	91	0.4	2
AVG/SUM	**8.85**	**192.97**	**15.74**	**20.86**	**9.49**	**88**	**6.7**	**4.09**

Table S3. Climatic data for the 10 days before harvest date (second year- 2021 experiments)

Date	Thermal amplitude	Radiation cal/cm2/dia	Mean temperature (°C)	Max T (°C)	Min temperature (°C)	RH (%)	Rainfall (mm)	Heliophany (h)
2021-10-24	12.1	639.3	19.3	24.7	10.6	56	0	11.9
2021-10-23	6.6	356.3	17.7	26.4	15.0	81	12.0	4.0
2021-10-22	16.2	631.8	21.5	28.2	10.0	54	5.4	11.8
2021-10-21	14.1	647.6	20.7	27.0	11.3	63	0	12.3
2021-10-20	15.0	635.0	20.4	27.1	10.0	62	0	12.0
2021-10-19	16.5	625.9	19.4	27.0	8.3	58	0	11.8
2021-10-18	16.1	616.8	18.3	26.2	6.8	58	0	11.6
2021-10-17	15.1	618.3	16.3	23.4	5.6	65	0	11.7
2021-10-16	13.0	626.7	14	20.6	6.0	62	0	12.0
2021-10-15	7.9	394.2	14.9	20.0	12.1	66	0.8	5.4
**AVG/SUM**	**13.26**	**579.19**	**18.25**	**25.06**	**9.57**	**62.5**	**18.2**	**10.45**
2021-09-21	8.4	510.5	14.1	18.6	8.5	65	0	10.4
2021-09-20	5.9	230.5	19.4	24.7	18.3	85	4.6	1.7
2021-09-19	9.2	222.6	20.5	24.8	14	84	0.4	1.5
2021-09-18	12.8	495.9	19.9	26,0	12	75	0	10.2
2021-09-17	15,0	464.7	17.6	25,0	8.3	72	0	9.3
2021-09-16	17.7	487.3	15.3	23.6	3.8	59	0	10.1
2021-09-15	14.6	493.7	13.3	20.1	3.2	62	0	10.4
2021-09-14	11.3	466,0	15.8	20.4	6.4	68	0	9.6
2021-09-13	8.1	407.7	22.7	29.3	14.6	87	0.7	7.8
2021-09-12	14.6	463.7	22.3	29.4	6,0	71	0	9.7
**AVG/SUM**	**11.76**	**424.26**	**18.09**	**24.19**	**9.51**	**72.8**	**5.7**	**8.07**
2021-08-23	1.8	140.7	14	18.2	11.6	60	0	0
2021-08-22	9.7	292.4	18.6	23.7	14	77	0	5.6
2021-08-21	11.1	211.6	15.4	20	7.6	75	0	2.7
2021-08-20	10	245	14.7	20	11.5	66	0	4
2021-08-19	16.4	227.1	22.8	31.6	15.2	69	0	3.4
2021-08-18	14	348.1	23.2	31.5	10.3	74	0	8
2021-08-17	16.9	356.4	18	26.7	8.4	78	0	8.4
2021-08-16	13.9	385.6	14.5	21.8	3.9	71	0	9.6
2021-08-15	13.7	301.7	11.9	20.1	3.2	79	0	6.5
2021-08-14	14.8	362.1	13.6	20.6	4	79	0	8.9
**AVG/SUM**	**12.23**	**287.07**	**16.67**	**23.42**	**8.97**	**72.8**	**0**	**5.71**
2021-07-28	7.5	307.5	7.7	13	2.9	68	0	8.2
2021-07-27	10.3	329.4	9.5	14.3	1.8	51	0	9.2
2021-07-26	7.4	233.7	14.7	17.8	13.2	75	0	5.2
2021-07-25	9.7	264.9	19.3	24.7	15	83	0.2	6.6
2021-07-24	11.6	203.1	19.5	25	12	67	2.5	4
2021-07-23	14.7	312.7	18.5	26.2	10.1	65	0	8.8
2021-07-22	15.8	290.3	14.7	23.2	5.7	69	0	7.9
2021-07-21	15.8	297.9	10.8	18.7	0.8	73	0	8.3
2021-07-20	13.4	312.3	9.3	16	0.2	72	0	9
2021-07-19	10.7	270	8.2	14	0.9	74	0	7.2
**AVG/SUM**	**11.69**	**282.18**	**13.22**	**19.29**	**6.26**	**69.7**	**2.7**	**7.44**
2021-06-29	6.4	196	7.6	9.9	2.5	78	0	4.6
2021-06-28	5.4	97.7	6.8	7	2.6	84	0	0
2021-06-27	7.1	244.4	9.2	11.1	4	73	0	6.9
2021-06-26	7.2	244.1	11.7	14.2	8	89	0	6.9
2021-06-25	12	190.8	11.1	17	3.6	90	0	4.4
2021-06-24	4.5	107.9	14.1	16.2	13.1	94	0.9	0.5
2021-06-23	2.5	97.2	15.1	15.8	12.1	93	82.3	0
2021-06-22	11.8	114.2	10.6	15.3	2	93	0	0.8
2021-06-21	7.4	156.6	13.2	16.9	4.8	81	0	2.8
2021-06-20	6.5	114.2	10.2	13.5	4.2	93	0	0.8
**AVG/SUM**	**7.08**	**156.31**	**10.96**	**13.69**	**5.69**	**86.8**	**83.2**	**2.77**

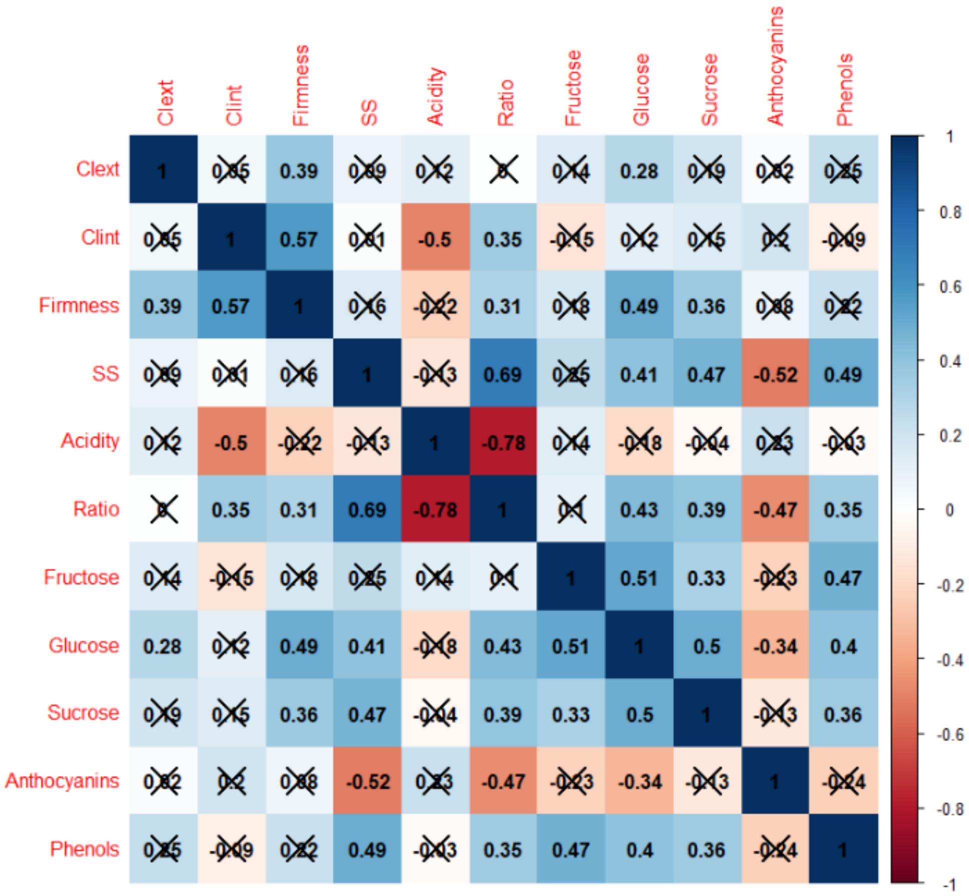

Supplementary Figure S1. Matrix of correlations for genotypes behavior study (data from all genotypes and harvest dates).

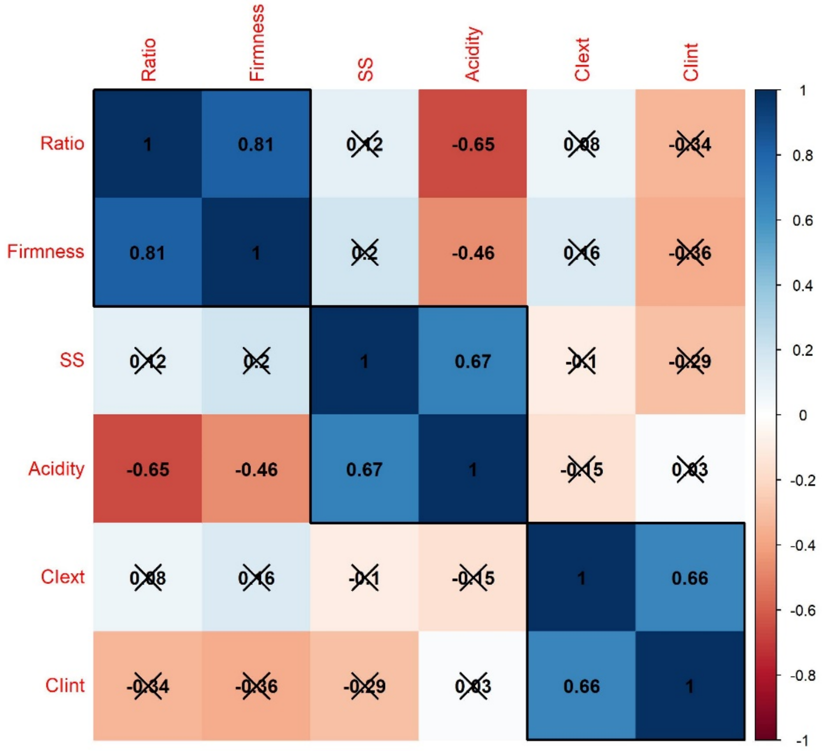

Supplementary Figure S2. Correlations in quality variables (Ratio, Firmness, Soluble solids-SS, Acidity, external color index- CIext and internal color index- CIint) for INIA Yrupé study.

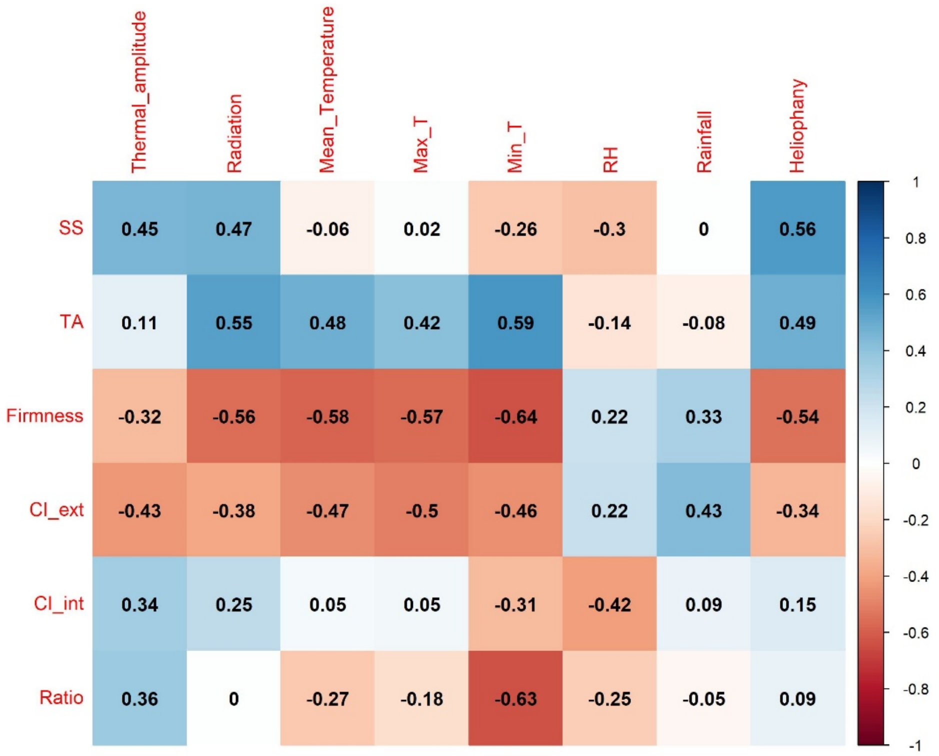

Supplementary Figure S3. Correlations between quality (Ratio, Firmness, Soluble solids-SS, Acidity-TA, external color index- CIext, internal color index- CIint) and environmental variables (Max, Min and Mean temperature, Heliophany, Rainfall, Relative humidity-RH, Radiation and Thermal amplitude) for INIA Yrupé study.
